# Biomarkers in prostate cancer: current status and future directions in radiotherapy—statement from the Prostate Cancer Working Group of the German Society of Radiation Oncology (DEGRO)

**DOI:** 10.1007/s00066-025-02388-x

**Published:** 2025-03-25

**Authors:** S. K.B. Spohn, D. M. Aebersold, C. Albrecht, D. Boehmer, U. Ganswindt, N.-S. Schmidt-Hegemann, S. Hoecht, T. Hölscher, S. A. Koerber, A.-C. Mueller, P. Niehoff, J. C. Peeken, M. Pinkawa, B. Polat, M. Shelan, F. Wolf, C. Zamboglou, D. Zips, T. Wiegel

**Affiliations:** 1 Department of Radiation Oncology, Medical Center – University of Freiburg, Faculty of Medicine, University of Freiburg, Robert-Koch-Straße 3, 79106 Freiburg, Germany; 2 Department of Radiation Oncology, Inselspital, Bern University Hospital, University of Bern, Freiburgstraße 4, 3010 Bern, Switzerland; 3 Nordstrahl Radiation Oncology Unit, Nürnberg North Hospital, Prof.-Ernst-Nathan-Str. 1, 90149 Nürnberg, Germany; 4 Charité—Universitätsmedizin Berlin, corporate member of Freie Universität Berlin and Humboldt-Universität zu Berlin, Klinik für Strahlentherapie, Hindenburgdamm 30, 12203 Berlin, Germany; 5 Department of Radiation Oncology, University Hospital Innsbruck, Anichstraße 35, 6020 Innsbruck, Austria; 6 Department of Radiation Oncology, LMU Munich, Marchioninistraße 15, 81377 Munich, Germany; 7 Department of Radiation Oncology, Ernst von Bergmann Hospital Potsdam, Charlottenstraße 72, 14467 Potsdam, Germany; 8 Department of Radiotherapy and Radiation Oncology, Faculty of Medicine and University Hospital Carl Gustav Carus, Technische Universität Dresden, Fetscherstr. 74, 01307 Dresden, Germany; 9 Department of Radiation Oncology, Heidelberg University Hospital, Im Neuenheimer Feld 400, 69120 Heidelberg, Australia; 10 Department of Radiation Oncology, Barmherzige Brüder Hospital Regensburg, Prüfeninger Straße 86, 93049 Regensburg, Germany; 11 Department of Radiation Oncology, RKH Hospital Ludwigsburg, Posilipostraße 4, 71640 Ludwigsburg, Germany; 12 Department of Radiation Oncology, Sana Hospital Offenbach, Starkenburgring 66, 63069 Offenbach, Germany; 13 Department of Radiation Oncology, TUM School of Medicine and Health, Technical University of Munich, Munich, Germany; 14 Department of Radiation Oncology, WEGE Klinik, Villenstraße 8, 53129 Bonn, Germany; 15 Department of Radiation Oncology, University Hospital Würzburg, Josef-Schneider-Straße 11, 97080 Würzburg, Germany; 16 Department of Radiation Oncology, Paracelsus University Hospital Salzburg, Müllner Hauptstraße 48, 5020 Salzburg, Austria; 17 German Oncology Center, European University of Cyprus, 1 Nikis Avenue, 4108 Agios Athanasios, Cyprus; 18 Charité—Universitätsmedizin Berlin, corporate member of Freie Universität Berlin and Humboldt-Universität zu Berlin, Klinik für Strahlentherapie, Augustenburger Platz 1, 13353 Berlin, Germany; 19 Department of Radiation Oncology, University Hospital Ulm, Albert-Einstein-Allee 23, 89081 Ulm, Germany

**Keywords:** Biomarkers, Prostate cancer, Radiotherapy, Precision medicine, Artificial intellegence

## Abstract

**Purpose:**

Prostate cancer (PCa) is the most frequently diagnosed malignancy among men in Germany. Advances in diagnostics and treatment have transformed PCa into a chronic disease. Given the heterogeneity of PCa, there is a need for additional stratification tools. This review focuses on updating the evidence for genomic classifiers (GC; Decipher [Veracyte Inc. San Diego, CA, USA], Prolaris [Myriad Genetics, Inc., Salt Lake City, UT], and Oncotype DX [Exact Sciences, Madison, WI, USA] tests) and artificial intelligence (AI)-based digital histopathology biomarkers (ArteraAI Prostate Test) in the context of radiotherapy (RT) for PCa.

**Methods:**

The members of the Prostate Cancer Working Group of the German Society of Radiation Oncology (DEGRO) conducted an updated literature search on GCs and histopathological biomarkers in PCa, covering original articles published between January 2022 and February 2024 in the PubMed database.

**Results:**

In addition to previous reviews, 11 relevant studies were identified, of which nine studies analyzed biomarkers within prospective phase II or III trials. Eight trials focused on genomic biomarkers, of which three addressed GCs in primary localized PCa, three in recurrent PCa in the setting of salvage RT, and two in metastatic castration-sensitive PCa. In localized PCa, GCs could be validated in a retrospective analysis of randomized controlled trials. Additionally, three studies reported on AI-based histopathology biomarkers.

**Conclusion:**

Genomic classifiers and AI-based digital histopathology models might have superior prognostic and predictive value compared to established clinical and pathological parameters in localized, recurrent, and metastatic PCa. Despite promising results, prospective validation of these biomarkers in randomized trials remains limited. This review underscores the need for further prospective trials to confirm the usefulness of these biomarkers in PCa.

## Introduction

Prostate cancer (PCa) is the most frequently diagnosed malignancy of men in Germany. Advances in diagnostics and treatment, with improved cure rates in localized and metastatic stages, have transformed PCa into a chronic disease. Risk stratification and treatment decision-making currently still mostly depend on clinical and pathological parameters, combining clinical TNM stage, Gleason score, and the prostate-specific antigen (PSA) value. The introduction of molecular imaging with ligands targeting the prostate-specific membrane antigen (PSMA) has shifted diseases stages and challenges current guideline treatment recommendations. Particularly in radiotherapy (RT), this additional information could be used to guide personalized RT approaches considering the individual tumor geometry and anatomy [1–3]. Various studies have demonstrated that PCa is a very heterogeneous disease, even within one risk group. Consequently, additional risk stratification tools are warranted. In recent years, the biological characterization of PCa has come into focus and genomic classifiers or mRNA-based gene expression profiles from PCa tissue have been developed and validated in order to improve risk stratification. The most commonly used and commercially available profiles include the Decipher (Veracyte Inc. San Diego, CA, USA), Prolaris (Myriad Genetics, Inc., Salt Lake City, UT), and Oncotype DX (Exact Sciences, Madison, WI, USA) panels [4, 5]. Since most of these classifiers have been validated in patients treated with radical prostatectomy to guide adjuvant treatment decisions, a systematic review and international Delphi consensus has summarized the emerging and available data for genomic classifiers (GC) in RT and pointed out future perspectives [6]. Recently, a multimodal artificial intelligence (MMAI)-based model of digitalized histopathology has been validated as another powerful prognosticator and predictor of treatment outcomes [7]. This review aims to update the evidence on genomic and histopathology biomarkers in RT practice.

## Methods and materials

In addition to a previously conducted systematic review, an NCBI PubMed search was performed using the following search terms: “genomic classifiers” and/or “biomarkers” and/or “prostate cancer” and/or “radiotherapy” (SS). In addition, all members of the Prostate Cancer Working Group of the German Society of Radiation Oncology (DEGRO) were asked to propose relevant literature. Only peer-reviewed original articles published between January 2022 and February 2024 were considered. Articles needed to be available in full text and written in English.

## Results

The updated literature research identified eight original studies on genomic biomarkers and three studies on histopathological biomarkers in PCa patients in the setting of definitive and postoperative RT. Nine studies evaluated biomarkers within prospective phase II or phase III trials [7–15]. One study included patients treated within a prospective quality of life study [16]. The study by Ramotar et al. was conducted in a retrospectively collected patient cohort [17]. Three studies addressed GC in primary localized PCa, three studies in recurrent PCa and in salvage radiotherapy (sRT), and two studies in metastatic castration-sensitive PCa (mCSPC). Three studies were identified reporting on AI-based histopathology biomarkers using the ArteraAI Prostate Test in primary localized disease. Table 1 summarizes these articles.

**Table 1:** Summary of included articles on genomic classifiers and histopathology-based biomarkers in prostate cancer in the setting of radiotherapy

Authors	Biomarker	Disease stage	Endpoint(s)	Cohort details	Results
Dal Pra et al. [8]	Decipher* test	Early sRT due to biochemical recurrence after prostatectomy	Primary: BF Secondary: CP, time to hormone therapy	*N* = 226 Phase III trial SAKK 09/10. Dose-escalated sRT of 70 Gy vs. standard dose of 64 Gy. No ADT	GC was an independent prognostic factor for BF (HR 2.26, 95%CI 1.42–3.60), CF (HR 2.29, 95%CI 1.32–3.98), and use of hormone therapy (HR 2.99, 95%CI 1.55–5.76)
Nguyen et al. [9]	Decipher biopsy test	High-risk localized PCa	Primary: DM Secondary: PCSM, OS	*N* = 265 Phase III trials NRG/RTOG 9413, 9202, and 9902 High-risk PCa, definitive RT of 65–70.2 Gy ± WPRT + ADT (4–28 months)	GC was an independent prognostic factor for DM (HR per 0.1 unit 1.22, 95%CI 1.09–1.36), PCSM (HR 1.23, 95%CI 1.09–1.39), and OS (HR 1.12, 95% CI 1.05–1.20)
Spratt et al. [10]	Decipher biopsy test	Intermediate-risk localized PCa	Primary: disease progression (composite of BF, LF, DM, PCSM, and salvage treatment)	*N* = 215 Phase III trial NRG/RTOG 01–26 Definitive RT of 70.2 Gy or 79.2 Gy, no ADT	GC was an independent prognostic factor for disease progression (HR per 0.1 point increase 1.12, 95%CI 1.00–1.26). Patients with high GC score had a 21% difference in 10-year MFS between treatment arms (70.2 vs. 79.2 Gy)
Repka et al. [16]	PTEN and ERG abnormalities using ProstaVysion test	Low-, intermediate-, and high-risk localized PCa	bPFS and OS	*N* = 99 SBRT of 35–36.25 Gy or combined SBRT and IMRT (19.5 Gy in 3 Fx + 45 Gy in 25 Fx) ± ADT	Wildtype ERG patients had improved 5‑year bPFS of 94.1% vs. 72.4% (*p* = 0.018). Wildtype PTEN patients had improved bPFS (901.0% vs. 67.9%, *p* = 0.006) and OS (96.4% vs. 79.4%, *p* = 0.001)
Ramotac et al. [17]	Decipher test and unfavorable subpathologies (intraductal carcinoma (IDC) and cribriform architecture (CA))	Postoperative radiotherapy (sRT and aRT)	bPFS and MFS	For Decipher *n* = 104, total cohort *n* = 302	IDC/CA presence was independently associated with decreased bPFS (HR 1.68, 95%CI 1.08–2.61). GC risk group was an independent prognostic factor for bPFS (HR intermediate vs. high 0.45, 95%CI 0.21–0.95; HR low vs. high 0.33, 95%CI 0.15–0.76). In exploratory analyses combining GC and IDC/CA, only GC remained a significant prognosticator for bPFS
Bitting et al. [11]	Decipher test including subtyping classifier, PTEN loss, homologous recombination deficiency (HRD), and ADT response	Biochemical recurrence after prostatectomy	PFS	*n* = 31 Phase II trial (STREAM) sRT + ADT + enzalutamide	Luminal proliferating subtype had worse 3‑year PFS than luminal differentiated (19% vs. 89%). PTEN loss (HR 1.32, 95%CI 1.07–1.64) and HRD (HR 1.21, 95%CI 1.05–1.39) were associated with worse PFS; high ADT response signature scores were associated with improved PFS (HR 0.75, 95%CI 0.61–0.94)
Sutera et al. [12]	NGS of primary or metastatic site WNT pathway mutations	Metachronous omCSPC	Descriptive statistics rPFS and OS	*N* = 277 SOC treatment or surveillance/MDT within the phase II STOMP and ORIOLE trials omCSPC (<=5 lesions on conventional imaging or PET)	11.2% of patients harbored WNT pathway mutations. WNT pathway mutations were associated with visceral metastases (22.6% vs. 2.8%; *p* < 0.01) and lower likelihood of regional lymph node metastases (29% vs. 50.4%; *p* = 0.02). WNT pathway mutations were not associated with rPFS but were associated with worse OS in the total cohort (HR 3.87, 95%CI 1.25–12.00). In patients who received MDT, pathogenic WNT pathway mutations were not associated with worse OS
Sutera et al. [13]	RNA sequencing of primary site	Synchronous and metachronous mCSPC	Primary: differences in transcriptomic profiles between synchronous and metachronous mCSPC Secondary: OS, MFS	*N* = 252 Treated in CHAARTED, STOMP, and ORIOLE trials or treated at Ghent University and Johns Hopkins Hospital	Patients with synchronous disease had worse 5‑year OS and lower AR activity. Patients with synchronous (HR = 0.47, 95%CI 0.30–0.72) but not metachronous (HR = 1.37, 95%CI 0.50–3.92) disease had better OS with AR and non-AR combination therapy compared to monotherapy
Esteva et al. [7]	MMAI model of clinical data and digital histopathology from prostate biopsies	Low, intermediate-, and high-risk localized PCa	MFS, OS, BF, PCSM	*N* = 5654 Phase III trials NRT/RTOG 9202, 0498, 9413, 9910, and 0126 Definitive RT ± ADT (4–28 months)	Compared to NCCN risk stratification, the MMAI model improved prognostication of 5‑year MFS (AUC 0.84 vs, 0.74; *p* < 0.001), 5‑year BF (AUC 0.67 vs. 0.59; *p* < 0.001), 10-year PCSM (AUC 0.77 vs. 0.68; *p* < 0.001), and 10-year OS (AUC 0.65 vs. 0.59; *p* < 0.001)
Spratt et al. [14]	MMAI of clinical data and digital histopathology from prostate biopsies	Intermediate- and high-risk localized PCa	Primary: development and validation of a predictive model to identify patients who benefit from short-term ADT in addition to RT Secondary: MFS, PCSM	*N* = 5727 Phase III trials NRG/RTOG 9202, 9413, 9910, 0126, and 9408 (validation cohort) Definite RT ± WPRT ± ADT (4–28 months)	ADT significantly improved MFS in patients with a positive model. The AI-model is predictive for ADT in mostly intermediate-risk PCa
Ross et al. [15]	MMAI model of clinical data and digital histopathology from prostate biopsies	High-risk or locally advanced PCa	DM, PCSM	*N* = 318 Phase III NRG/RTOG 9902 trial RT + long-term ADT or with adjuvant chemotherapy	MMAI model was independently associated with a higher risk of DM (HR 2.33, 95%CI 1.6–3.38) and PCSM (HR 3.54, 95%CI 2.38–5.28) compared to clinical factors and NCCN risk features

*PCa* prostate cancer, *DM* distant metastases, *OS* overall survival, *omCSPC* oligometastatic castration-sensitive prostate cancer, *MFS* metastases-free survival, *HR* hazard ratio, *RT* radiotherapy, *ADT* androgen deprivation therapy, *AUC* area under the curve, *NCCN* national comprehensive cancer network, *MMAI* multimodal artificial intelligence, *bPFS* biochemical progression-free survival, *CP* clinical progression, *IDC/CA* intraductal cancer/cribriform architecture, *WPRT* whole-pelvic radiotherapy; *NGS* next-generation sequencing, *SOC* standard of care, *rPFS* radiographic progression-free survival, *AR* androgen receptor, *PCSM* prostate cancer-specific mortality, *Fx* fractions

* Decipher (Veracyte Inc.  San Diego, CA, USA)

## Discussion

Prostate cancer is a heterogeneous disease with varying outcomes depending on the disease stage. Whereas prostate cancer-specific mortality (PCSM) for localized disease is very low, with rates of less than 3% after 15 years [18], median overall survival for patients with metastatic disease can be less than 4 years [19]. Treatment options over the course of disease include RT or surgery and systemic treatment agents, particularly androgen deprivation therapy (ADT). In mCSPC, new hormonal agents such as apalutamide [20], enzalutamide [21], and abiraterone/prednisone [22] in combination with ADT and darolutamide in combination with ADT and docetaxel [19] improve OS compared to ADT or ADT/chemotherapy alone. Moreover, these intensified systemic treatments prolong the time to castration resistance, a disease stage associated with poor oncological outcomes [23]. Thus, treatment in earlier disease stages needs to be optimized in order to reduce the risk of transformation into the castration-resistant state. Current risk stratification systems for localized PCa incorporate clinical and pathological parameters to stratify patients from low to high risk with subsequent treatment recommendations. However, these models insufficiently reflect the aggressiveness of PCa. Novel biomarkers are warranted to identify patients at a higher risk of progression who may benefit from treatment intensification and patients at a lower risk who may be candidates for treatment de-intensification with less toxicity and improved quality of life.

Genomic profiling of PCa biopsy tissue can be performed using commercially available panels. The Decipher genomic classifier is the most commonly used test. Several retrospective studies have validated GC as independent prognostic factors for DM and biochemical recurrence after definitive RT in localized PCa [24–28]. We identified two additional studies retrospectively validating the Decipher GC—analyzed with the biopsy test—in phase III randomized controlled trials [9, 10]. These studies add higher-level evidence that GC can guide treatment decision-making in high- and intermediate-risk as well as recurrent PCa. Interestingly, the study from Spratt et al. also demonstrated a predictive value, with patients with high GC scores experiencing the maximum benefit from dose-escalated radiotherapy [10]. For localized PCa, the digital histopathology-based model by Artera (ArteraAI Prostate Test) has been validated as an independent prognostic biomarker. This model was initially trained using clinical and histopathology data from five randomized controlled trials [7]. This system comes with low barriers, since digitized histopathology data can easily be obtained and shared for deeper analysis, thus reducing turnaround times. Validation in low-, intermediate-, and high-risk as well as locally advanced disease demonstrated that the histopathology-based multimodal artificial intelligence (MMAI) model improves outcome prognostication significantly and meaningfully [7]. Moreover, Spratt and colleagues were also able to demonstrate in a retrospective validation that the MMAI biomarker has a predictive value and could identify patients who might benefit from short-term ADT in addition to RT [14].

These studies have shown that there are powerful biomarkers available that might improve risk stratification and could be used to guide treatment recommendations. Further clinical trials such as the ongoing PREDICT-RT trial (NRG-GU009, NCT04513717), which randomizes patients according to their Decipher genomic risk to ADT intensification (24 months of apalutamide and ADT for patients with NCCN high risk who are in the upper 1/3 of the Decipher genomic risk or have node-positive disease by conventional imaging) or de-intensification (12 months of ADT for patients with NCCN high risk who are in the lower 2/3 of Decipher genomic risk), will assess whether incorporating these novel biomarkers into treatment decision trees can minimize over- and undertreatment. So far, the available evidence is limited to retrospective analyses of prospective trials in the primary setting.

Patients who receive sRT due to biochemical recurrence or PSA persistence after prostatectomy experience heterogeneous outcomes depending on clinical and pathological features such as the PSA level prior to sRT [29]. The Decipher and Prolaris tests have been retrospectively validated as independent prognostic factors for various oncological outcomes after sRT [30, 31]. An analysis of the Decipher GC within the SAKK 09/10 phase III trial confirmed the improved prognostic value of this test [8]. We identified one additional study, confirming that adverse pathological subtypes such as intraductal carcinoma or cribriform architecture are associated with worse biochemical progression-free survival [17]. Interestingly, for this patient subgroup, GC added a significant prognostic value. Another study by Bitting and colleagues performed genomic profiling of 31 patients within the phase II STREAM trial that investigated the safety and efficacy of concurrent enzalutamide with sRT and ADT (38 enrolled patients in total) [32]. In addition to the Decipher GC, other mutations including PTEN loss, homologous recombination deficiency, and ADT response scores were significantly associated with progression-free survival (PFS). These patients with worse prognoses might particularly benefit from systemic treatment intensification in addition to sRT with new hormonal agents such as enzalutamide [33]. Ongoing trials include the NRG GU006 (BALANCE, NCT03371719) phase II trial, the RTOG 3506 (STEEL NCT03809000) phase II trial, and the Formula 509 (NCT03141671) phase II trial. Formula 509 investigates systemic treatment intensification with apalutamide and abiraterone/prednisolone in addition to ADT. In this setting, novel biomarkers, such as GC, might be a powerful tool to identify patients who could benefit from escalated systemic treatments in addition to sRT.

Several phase II trials have demonstrated potential benefits of adding metastasis-directed therapy (MDT) in oligometastatic disease [34–36], with the optimal treatment still subject to research. PCa experiences changes in the proteogenomic landscape during the course of disease [37], and metastatic PCa is a clinically and biologically heterogeneous disease. We found two studies by Sutera et al., who performed NGS of the primary and metastatic site and RNA sequencing of the primary site in synchronous and metachronous mCSPC. The first study showed that WNT pathway mutations were associated with worse outcomes [12]. Interestingly, in patients who received MDT, WNT pathway mutations were not associated with worse OS. A previously published study by Deek et al. also demonstrated a potential larger benefit for MDT in patients with high-risk mutations [38]. The second study demonstrated differences in OS and androgen receptor activity between synchronous and metachronous disease. Furthermore, synchronous disease was associated with better OS if AR and non-AR therapy was combined [13]. These data suggest that genomic profiling might improve patient selection for optimal systemic treatment and MDT. Future trials should incorporate biomarkers to fill the gap due to the lack of prospective data in this setting.

In summary, genomic classifiers and AI-based digital histopathology models might have superior prognostic value compared to classical clinical and pathological parameters and a predictive value in localized PCa. The growing number of high-quality studies support the clinical utilization of these novel biomarkers. Additional genetic profiling might also improve treatment selection for patients with recurrent and metastatic PCa. However, prospective validation of these tests is currently missing.

This is in large contrast to breast cancer, where several multigene tests have been validated in prospective trials [39–41]. Thus, German health care insurance providers currently reimburse genetic tests in breast cancer to guide personalized treatment decisions in terms of adjuvant chemotherapy, whereas PCa patients have to cover these costs privately if they have access to the respective facilities. Interestingly the NCCN guidelines in the US recommend using genomic tests as tools if they “have the potential to change disease management” in PCa (NCCN guidelines on prostate cancer, version 3.2024) and MEDICARE reimburses testing with Decipher and the ArteraAI Prostate Test in localized, advanced, and recurrent PCa. The current EAU guidelines are more reluctant and mention the prognostic value of as promising but demand further data before they can be used in standard clinical practise [42]. In order to generate the urgently needed prospective validation of genomic tests, relevant research needs to be promoted by stakeholders and politics in close collaboration with patient representatives. Furthermore, these biomarkers bear the potential to increase cost effectiveness through optimized treatment selection [43].
